# Frizzled-4 C-terminus Distal to KTXXXW Motif is Essential for Normal Dishevelled Recruitment and Norrin-stimulated Activation of Lef/Tcf-dependent Transcriptional Activation

**DOI:** 10.5334/1750-2187-11-1

**Published:** 2016-02-05

**Authors:** Alexander C. Bertalovitz, Milly S. Pau, Shujuan Gao, Craig C. Malbon, Hsien-yu Wang

**Affiliations:** Department of Physiology & Biophysics, Health Sciences Center, School of Medicine, Stony Brook University, Stony Brook, NY 11794-8661, USA; Department of Pharmacology, Health Sciences Center, School of Medicine, Stony Brook University, Stony Brook, NY 11794-8651, USA

**Keywords:** Frizzled, Frizzled-4, carboxy-terminus, helix VIII, Dishevelled, Norrin

## Abstract

The carboxy (C)-termini of G protein coupled receptors (GPCR) dictate essential functions. The KTXXXW motif C-terminus of Frizzleds (FZD) has been implicated in recruitment of Dishevelled (DVL). Through study of FZD4 and its associated ligand Norrin, we report that a minimum of three residues distal to the KTXXXW motif in the C-terminal tail of Frizzled-4 are essential for DVL recruitment and robust Lef/Tcf-dependent transcriptional activation in response to Norrin.

## Background

The carboxy-terminal tail (C-tail) of GPCRs plays an essential role in receptor function and biology [1]. Mediating interactions with chaperones and downstream signaling elements, the C-tail may play a role in cell surface expression and downstream signal transduction [2]. Agonists binding to GPCRs have been shown to induce changes in C-tail conformation necessary for activating heterotrimeric G protein [3]. Prolonged agonist stimulation catalyzes phosphorylation of the C-tail, promoting arrestin binding, desensitization, and ultimately GPCR internalization [1].

Short amphipathic membrane interacting helix VIII at the transmembrane domain 7 (TM7) proximal end of the C-tail of GPCRs has been shown to be critical for G protein coupling and receptor trafficking [4,5]. Although not reported for FZD, a crystal structure exists for the smoothened receptor [6]. To facilitate efforts in crystallization portions of the C-tail of GPRCs are often times truncated [7,8]. For smoothened receptor, the flexibility of long unstructured regions of the C-tail required truncation at Q555, removing 200+ residues of the C- tail. The helix VIII was found to extend two residues beyond the tryptophan corresponding to that of FZD KTXXXW domains. Studies utilizing peptides encoding the C-tail of FZD suggest alpha-helicity and some role of the region immediately distal to KTXXXW in facilitating a receptor interaction with the PDZ domain of DVLs [9,10].

Reports from studies employing full-length FZDs are not uniform concerning the function of the KTXXXW motif. Perturbation of the KTXXXW motif appears to inhibit the ligand-independent colocalization of Xenopus FZD_3_ with DVL [11]. Drosophila FZD_2_ lacking the KTXXXW motif, in sharp contrast, exhibited robust activation of Wnt/Wingless-induced Lef/Tcf-dependent transcription [12]. Promiscuity of various Wnts for Frizzleds occurs [13]. These possible promiscuous interactions are compounded at super physiological stoichiometry of FZD expressed in various cell lines [14]. These are formidable stumbling blocks to fine structure-activity analyses of FZD. To obviate these issues and probe the C-tail of FZD we took advantage of the FZD_4_-Norrin specificity [15,16]. We found that the three residues QKC distal to the highly conserved KTXXXW domain of the FZD_4_ are required for substantial DVL recruitment and Lef/Tcf-dependent transcriptional activation.

## Materials and Methods

### Construction of plasmids

The mouse FZD_4_ construct containing the V5-tag between F37 and G38 in a prk5 vector (Addgene, Cambridge, MA) was used to generate mutants employed in this study. The QuikChange Site-Directed Mutagenesis Kit (Agilent, Santa Clara, CA) was employed with specific primers to mutate codons. To construct the mFZD_4_/C-tailmFZD_1_, mFZD_4_/C-tailmFZD_3_, and the mFZD_4_/C-tailmFZD_7_ chimeras in which the C-tail after the KTXXXW of mFZD_4_ was substituted with the corresponding region from mFZD_1_, mFZD_3_, or mFZD_7_, respectively, the overlap extension polymerase chase reaction (PCR) method was employed using the Phusion Hot Start II DNA polymerase (Thermo Scientific, Waltham, MA). The C-terminal green fluorescent protein (GFP)-tagged DVL2 construct was generated by inserting the Human DVL2 gene into the pEGFPN vector (Clontech, Mountain View, CA). The constructs were verified by DNA sequencing.

### Cell culture

Human embryonic kidney (HEK293) and HeLa cells (obtained from ATCC, Manassas, VA) were cultured in Dulbecco’s modified Eagle’s medium (Cellgro, Manassas, VA) supplemented with fetal bovine serum (10%, Hyclone, South Logan, UT), penicillin (100µg/ml) and streptomycin (100µg/ml, Corning, Manassas, VA) in a humidified atmosphere with a 5% CO_2_ level at 37°C.

### Lef/Tcf-dependent transcriptional activation via luciferase reporter assays

HEK293 cells were cultured in gelatin-coated 96 well plates (Greiner Bio-One, Frickenhausen, Germany) and then transfected at ~75% confluency using Lipofectamine 2000 (Life Technologies, Carlsbad, CA). Briefly, typical transfection conditions were as follows: functional assays included 0.5 ng of receptor, 1 ng of hLRP5 co-receptor, 10 ng of Super8xTOPFlash (M50) and pcDNA3.1 empty vector to a total of 50 ng plasmid DNA per well. Cells were stimulated with recombinant Norrin (200 ng/mL, R&D Systems, Minneapolis, MN), LiCl (50 mM) or left unstimulated for ~18 h 24 h after transfection. Lysis of cells was performed with the cell culture lysis buffer (Cat# E153A, Promega, Madison, WI). Cell lysates (20 µl) were added to 100 µl of luciferase assay buffer (20 mM Tricine pH 7.8, 1.1 mM MgCO_3_, 4 mM MgSO_4_, 0.1 mM EDTA, 0.27 mM coenzyme A, 0.67 mM luciferin, 33 mM DTT and 0.6 mM ATP). The luminescence intensity was measured with a Lumat LB 9507 luminometer (Berthold Technologies, Oak Ridges, TN). Conditions were performed minimally in triplicate, unless otherwise stated in the figure legend. Bar graphs display the % wild-type (WT) activation for Norrin-treated cells. The error bars display the standard error of the mean (S.E.M.). To determine the % WT activation the RLU values for each Norrin-stimulated condition were first divided by the mean LiCl stimulation RLU values for the same condition determining the % maximal stimulation value for each Norrin-stimulated sample. The mean % maximal stimulation value for the WT Norrin-stimulated samples was set as 100% WT activation.

### Confocal microscopy

HeLa cells were cultured in 4 compartment CELLVIEW^TM^ glass-bottom dishes (Greiner Bio- One, Frickenhausen, Germany) and transfected at ~75% confluency with Lipofectamine 2000. Briefly, the conditions were as follows: 10 ng of GFP-tagged DVL2, 10 ng of Frizzled and empty vector to 250 ng per quadrant. Approximately 40 h after the transfections HeLa cells were then fixed with 4% paraformaldehyde (Electron Microscopy Sciences, Hatfield, PA) at room temperature following the removal of the media. Fixed cells were then washed 3X with Hank’s Balanced Salt Solution (HBSS, Life Technologies, Carlsbad, CA). Following the third wash, the quadrants were incubated with two drops of Image iT-FX signal enhancer (Molecular Probes, Eugene, Oregon) and rocked for 30–60 min at room temperature. Cells were then washed with HBSS 3X prior to the addition of V5 antibody (Novex, Carlsbad, CA at 1:1000) diluted in 2% sterile filtered fraction V bovine serum albumin (BSA), (MP Biomedicals, Santa Ana, CA) containing HBSS solution overnight at 4°C. The following day the cells were washed 3X with HBSS and then incubated with Alexa Fluor 594 labeled secondary antibody (Molecular Probes, Eugene, Oregon) for 90 min at room temperature in the dark. Cells were washed again and maintained in HBSS at 4°C in the dark until Fluorescent and differential interference contrast (DIC) images were taken using a FluoView FV1000 confocal laser scanning microscope (Olympus, Tokyo, Japan) with a 60X oil immersion objective lens.

### Receptor surface expression measured by IFA

Immunofluorescence assay (IFA) transfections were performed similarly to those of the functional assays, with 2 ng of receptor DNA per well. 24 h after, HEK293 cells were plated on gelatin-coated black plate clear bottom 96 well assay plates (Corning Inc., Corning, NY) in at least duplicate and fixed with 4% paraformaldehyde. Cells were subsequently washed with HBSS then blocked with 2% BSA. The cells were incubated with V5 antibody (Novex, Carlsbad, CA) at a 1:500 dilution, then washed with HBSS and incubated thereafter with Alexa Fluor 594 anti-mouse antibody diluted 1:1500. Cells were washed with HBSS and a SpectraMax M5 multimode plate reader (Molecular Devices, Sunnyvale, CA) was used to determine fluorescence readings for each well. Bar graphs display the % WT surface expression for each condition with the error bars representing the S.E.M. To determine the % WT surface expression for each condition the mean RFU value from the wells transfected without Frizzled plasmid was first subtracted from the other values. The mean of the resulting WT surface expression values was set as 100% WT surface expression.

### Data analysis

To test for statistical significance between data, unpaired *t*-tests or one-way analysis of variance (ANOVA) followed by the Dunnett’s post test were conducted as described in the figure legends. *p* values < 0.05 established statistical significance.

## Results

**Figure 1:**
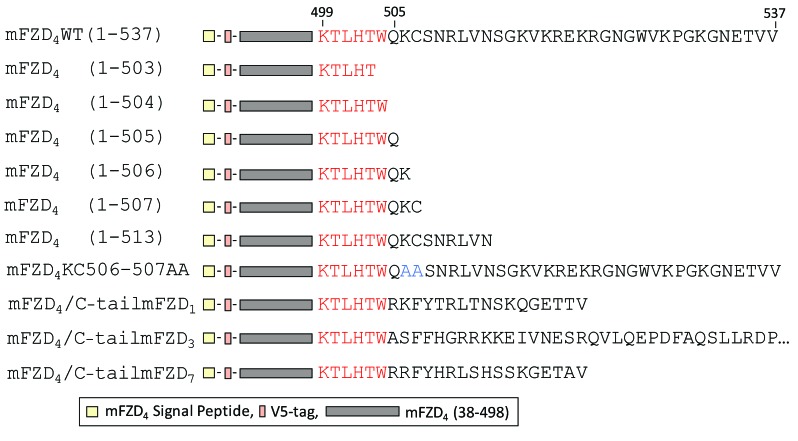
**Schematic representation of the FZD_4_ C-tail variants investigated in this study.** The constructs were generated using a template containing a V5 tag inserted in the amino- terminus of the protein following the signal peptide. The residues of the highly conserved KTXXXW domain are colored red. The alanine residues substituted for the lysine and cysteine residues in the mFZD_4_KC506-507AA receptor are colored blue. The mFZD_4_/C-tailmFZD_1_, mFZD_4_/C-tailmFZD_3_, and mFZD_4_/C-tailmFZD_7_ constructs differ from the mFZD_4_ WT construct. In these chimera, the region of the C-tail distal to the KTXXXW domain of mFZD_4_ has been substituted by the corresponding region of mFZD_1_ (mFZD_1_ residues R626 to V642), mFZD_3_ (mFZD_3_ residues A508 to A666 of mFZD_3_) or mFZD_7_ (mFZD_7_ residues R556 to V572), respectively. Note that for simplicity the mFZD_4_/mFZD_3_ C-tail is not shown in its entirety.

**Figure 2:**
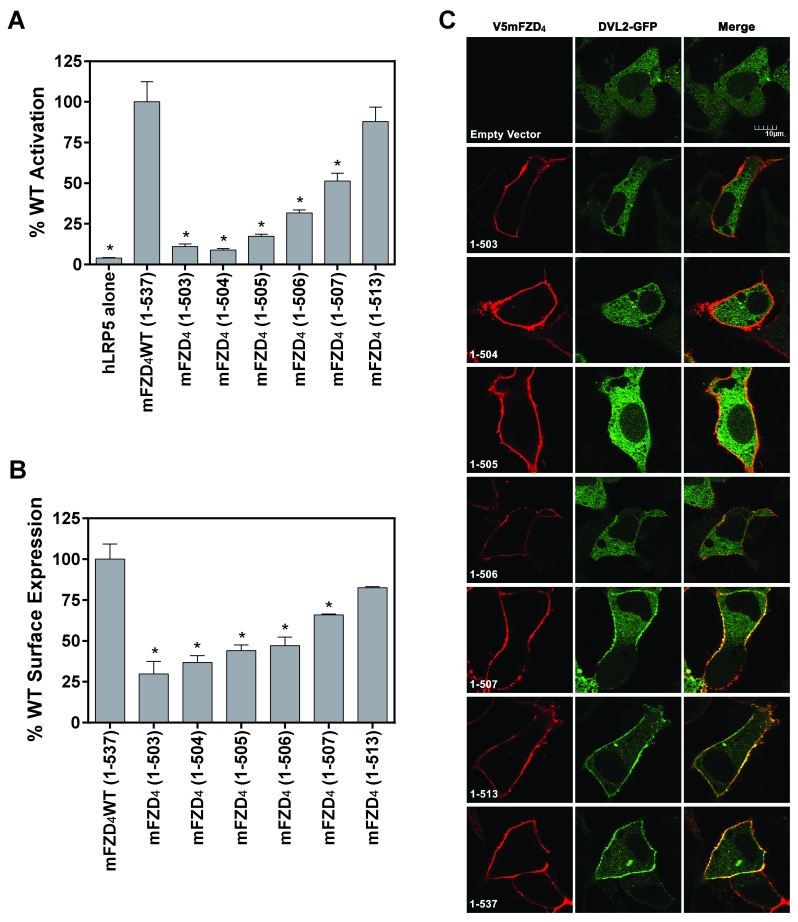
**The FZD_4_ C-tail modulates surface expression and receptor-DVL interaction.** (A) V5mFZD_4_ variants were overexpressed in HEK293 cells with hLRP5 and M50 reporter plasmid, stimulated with Norrin and Lef/Tcf-dependent luciferase activity was assayed as described. (B) Cell surface receptor expression detected by IFA in HEK293 cells transfected with the V5mFZD_4_ variant shown and hLRP5. Following fixation the non-permeabilized cells were incubated with V5 primary antibody followed by incubation with a compatible Alexafluor594 secondary antibody and subsequent fluorescence quantification on a multimode plate reader. Statistically significant differences compared to WT as determined by an ANOVA analysis followed by the Dunnett’s *post hoc* test is indicated with an asterisk (*). (C) Confocal images of non-permeabilized HeLa cells co-transfected with GFP-tagged DVL2 and V5mFZD_4_ WT or truncation variants using a V5 primary antibody.

We analyzed the effects that truncation and substitution of the mouse Frizzled-4 C-tail (Figure 1) have on the ability of Norrin to induce Lef/Tcf-dependent transcriptional activation (Figure 2A). Mutant mFZD_4_ (1-503) truncates the C-tail immediately beyond T503. Truncation at this position (mFZD_4_(1-503)) nearly abolished the ability of Norrin to activate Lef/Tcf- dependent transcription (Figure 2A). Restoring the wild-type sequence of the C-tail beyond T503 to N513 (mFZD_4_(1-513)) gradually ameliorated the loss of the Lef/Tcf-dependent transcriptional activation in response to Norrin to that of the WT response. We gauged the expression of mFZD_4_ and the truncations using amount of the surface expression (Figure 2B). All of the FZD_4_ constructs were expressed. Truncating the FZD_4_ carboxy-terminus at W504 to S508 resulted in some loss of cell surface expression. Although a positive correlation was observed between C-tail length and surface expression, the loss in activation was far greater than the apparent loss of some surface expression, suggesting that the simple reduction in cell surface mFZD_4_ could not account for the more profound loss of its function.

We probed the possibility that the changes in downstream signaling of the truncated mFZD_4_ might be accessible at the level of DVL (Figure 2C). We interrogated the ability of the mFZD_4_ and its variants to stabilize DVL at the cell surface. To enable these studies we made use of GFP-tagged DVL2, which is often times the most abundant DVL [17]. The ability of Frizzleds to stabilize DVL at the cell surface is essential to beta-catenin-dependent activation of the downstream pathway leading to Lef/Tcf-dependent transcriptional activation [11,18]. stabilization by FZD can be readily detected by confocal imaging of non-permeabilized cells expressing GFP-tagged DVL2 and V5-tagged mFZD_4_ (Figure 2C). We were unable to detect significant DVL2 recruitment and stabilization by mFZD_4_ C-tail mutants mFZD_4_(1-503), mFZD_4_(1-504), or mFZD_4_(1-505). These Frizzled mutants were unable to promote readily detectable DVL2 stabilization at the cell surface. mFZD_4_ truncated at C507 (mFZD_4_ (1-506)) exhibited subtle punctate accumulation of DVL2 at the cell surface, but to a markedly lesser degree than the WT mFZD_4_. Extending the C-tail of mFZD_4_ at least three residues beyond the KTXXXW motif, e.g., mFZD_4_ (1-507) or mFZD_4_(1-513), largely reversed this loss-of-function (Figure 2A) and substantial DVL recruitment was observed (Figure 2C).

**Figure 3:**
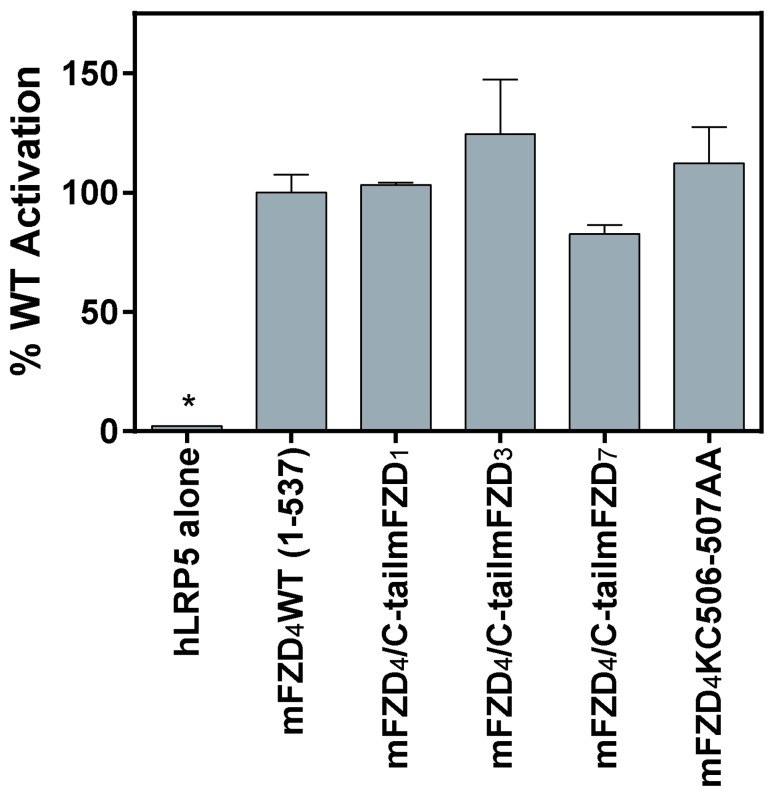
**The FZD_4_ C-tail distal to the KTXXXW domain requires three additional amino acid residues for substantial activation of Lef/Tcf-dependent transcriptional activation by Norrin.** V5mFZD_4_ variants were overexpressed in HEK293 cells with hLRP5 and M50 reporter plasmid, stimulated with Norrin. Lef/Tcf-dependent transcriptional activation was measured using a luciferase-based assay, as described in the *Materials and Methods* section. An experiment comparing the Norrin-induced mFZD_4_ WT activation to the Norrin-induced activation of the mFZD_4_/C-tailmFZD_1_, mFZD_4_/C-tailmFZD_3_, mFZD_4_/C-tailmFZD_7_ and mFZD_4_ KC506-507AA constructs. Statistically significant differences compared to WT as determined by an ANOVA analysis followed by the Dunnett’s *post hoc* test are indicated with an asterisk (*).

To determine if the specific side chain properties of either K506 or C507 are required for receptor activation a double mutant was generated in which K506 and C507 were concurrently substituted with alanine residues (Figure 1). This mFZD_4_KC506-507AA mutant exhibited normal Norrin-induced Lef/Tcf-dependent transcriptional activation (similar to WT, see Figure 3). The mFZD_4_KC506-507AA double mutant also stabilized DVL2 at the cell surface (data not shown).

Despite reports that under specific conditions FZD_3_ can mediate beta-catenin-dependent signaling [11,19] FZD_3_ and FZD_6_ have been reported to induce the least amount of Lef/Tcf- dependent activation amongst the 10 Frizzleds following the addition of various Wnts [13,20]. With 159 amino acids following the KTXXXW domain of FZD_3_ (the corresponding region of FZD_4_ has 33 amino acids) we hypothesized the relatively long FZD_3_ C-tail might have a role inhibiting or precluding FZD_3_-mediated Lef/Tcf-dependent activation. A construct was generated in which the mFZD_4_ region after the KTXXXW domain was replaced with the corresponding region from mFZD_3_ (Figure 1). The mFZD_4_ C-tail was also substituted with the corresponding region from mFZD_1_, and mFZD_7_, two Frizzleds which in many systems mediate more robust Tcf/Lef-dependent activation than FZD_3_ [13,20]. The Norrin-induced activation of Tcf/Lef-mediated gene transcription for all three chimeric receptor constructs was similar to that of WT mFZD_4_ (Figure 3). Interestingly, the mFZD_4_/C-tailmFZD_3_ chimeric receptor exhibited enhanced basal activity (Additional file 1).

## Discussion

In this study we show the C-tail of FZD_4_, beyond the KTXXXW domain, affects several aspects of Frizzled-4 signaling and biology. The crystal structure of the smoothened receptor displays the region of the C-tail with a sequence highly homologous to the KTXXXW motif of the Frizzleds [6]. This domain has a short helix VIII parallel to the membrane encompassing the lysine extending to two residues beyond the tryptophan of the motif. A circular dichroism spectroscopy analysis of a peptide consisting of the FZD_4_ C-tail in conjunction with molecular modeling suggests a helix VIII extending six residues beyond the KTXXXW motif of FZD_4_ [10]. In a membrane-mimicking environment a FZD_1_ C-tail peptide exhibited a helix encompassing the leucine of the KTXXXW motif to 9 residues after the tryptophan [21]. Interestingly, the tryptophan residue, corresponding to W504 of mFZD_4_, was shown to interact with the artificial lipid bilayer suggesting it could mimic the role of a C-tail palmitoyal group which has been demonstrated to stabilize the helix 8 of various GPCRs [22,23]. The mFZD_4_KC506-507AA mutation in which the only cysteine in the C-tail was substituted with alanine exhibited normal Norrin-induced Lef/Tcf-dependent transcription signifying the absense or lack of a role in Lef/Tcf-dependent transcription of a palmitoylation site in the C-tail of FZD_4_.

Other results obtained using peptides that map to the C-termini of Frizzleds suggest some function extending five to nine residues following the KTXXXW motif of FZD_5_ and FZD_7_. Productive interaction between the FZD_5/7_ with the DEP and PDZ domains of DVL appear to be influenced by this region of FZD [9,24]. The present study indicates functionality that extends beyond the KTXXXW motif by more than three residues. The QKC residues were shown to ameliorate receptor trafficking and Lef/Tcf-dependent transcription as a series of receptor constructs were generated including more of these residues. Although, WT-like signal transduction and trafficking observed with mFZD_4_(1-513) was not detected with mFZD_4_(1-507) signifying residues distal to QKC may also have a role in enabling the formation of a helical structure critical for the life-cycle of the receptor.

Shortening the C-tail beyond C507 severely impaired normal DVL recruitment (as observed by FZD-DVL colocalization) and the ability of Norrin to activate Lef/Tcf-dependent transcription. The intracellular loops (iloops) of Frizzleds also interact with DVL [18,24]. It is possible that the Frizzled C-tail provides additional but essential interactions with DVL required for the receptor to participate in normal DVL recruitment and stabilization.

Multiple Frizzleds have been shown to mediate Wnt-induced activation of Lef/Tcf- dependent transcription to differing extents [20]. FZD_3_ and FZD_6_, for example, appear to activate Lef/Tcf-dependent transcription less robustly than FZD_1_ or FZD_7_ [13,25]. The C-tails of FZD_3_ and of FZD_6_ are considerably longer than those of other Frizzleds. Substituting the corresponding C-tail of FZD_4_ distal to the KTXXXW sequence with that from FZD_1_, FZD_3_, or FZD_7_ did not impact the ability of these mutant versions of FZD_4_ to mediate Norrin-induced Lef/Tcf-dependent transcriptional activation. The FZD_1_, FZD_3_, and FZD_7_ C-tails may provide a structural role like that of FZD_4_’s own native C-tail, *i.e.*, any secondary structure distal to transmembrane domain 7, such as helix VIII of FZD_4_, may form in the corresponding region of these other Frizzleds. The reduced ability of specific receptors to activate Lef/Tcf-dependent transcription may be due to factors outside of the ability of the C-tail to interact with DVL such as the potential of the receptor to synergize with LRP5/6 following the binding of a WNT ligand or other differences resulting from variations in the core of the receptors.

In summary, three residues distal to the KTXXXW motif of FZD_4_ are essential to normal FZD_4_-DVL interactions that are required for Lef/Tcf-dependent transcriptional activation by Norrin. Alpha-helicity in this region of the Frizzleds seems obligate for efficient protein-protein interaction with DVL and other downstream signaling elements.

## Conclusion

This study demonstrates that substantial function of the FZD_4_ C-terminal tail minimally requires the three residues distal to the conserved KTXXXW domain. These additional residues QKC participate in cell-surface expression of Frizzled-4 and for signal propagation via Frizzled- DVL interactions that enable Norrin-dependent activation of Lef/Tcf-dependent transcription.

## Competing Interests

The authors declare that they have no competing interests.

## Abbreviations

ANOVA: analysis of variance

BSA: bovine serum albumin

C-tail: carboxy-terminal tail

C- termini: carboxy-termini

DIC: differential interference contrast

DVL: Dishevelled

FZD: Frizzled

GFP: green fluorescent protein

HBSS: Hank’s Balanced Salt Solution

HEK293: human embryonic kidney

IFA: immunofluorescence assay

iloops: intracellular loops

M50: Super8xTOPFlash

mFZD: mouse Frizzled

PCR: polymerase chase reaction

RFU: relative fluorescence units

RLU: relative light units

S.E.M.: standard error of the mean

WT: wild-type
